# The role of LDH and ferritin levels as biomarkers for corticosteroid dosage in children with refractory *Mycoplasma pneumoniae* pneumonia

**DOI:** 10.1186/s12931-024-02892-1

**Published:** 2024-07-04

**Authors:** DiWei Wei, YiDi Zhao, TongQiang Zhang, YongSheng Xu, Wei Guo

**Affiliations:** 1 Department of Pulmonology, Tianjin Children’s Hospital (Children’s Hospital of Tianjin University), Tianjin Pediatric Research Institute and Tianjin Key Laboratory of Birth Defects for Prevention and Treatment, Tianjin, 300134 China; 2 Children’s Clinical College of Tianjin Medical University, Tianjin, China

**Keywords:** *Mycoplasma pneumoniae*, Lactate dehydrogenase, Ferritin, Glucocorticoids, Children

## Abstract

**Background:**

This study explored the relationship between inflammatory markers and glucocorticoid dosage upon admission.

**Methods:**

We conducted a retrospective analysis of 206 patients with refractory *Mycoplasma pneumoniae* pneumonia (RMPP) admitted to a Children’s Hospital from November 2017 to January 2022. Patients were categorized into three groups based on their methylprednisolone dosage: low-dose (≤ 2 mg/kg/d), medium-dose (2–10 mg/kg/d), and high-dose (≥ 10 mg/kg/d). We compared demographic data, clinical manifestations, laboratory findings, and radiological outcomes. Spearman’s rank correlation coefficient was used to assess relationships between variables.

**Results:**

The median age was highest in the low-dose group at 7 years, compared to 5.5 years in the medium-dose group and 6 years in the high-dose group (*P* < 0.001). The body mass index (BMI) was also highest in the low-dose group at 16.12, followed by 14.86 in the medium-dose group and 14.58 in the high-dose group (*P* < 0.001). More severe radiographic findings, longer hospital stays, and greater incidence of hypoxia were noted in the high-dose group (*P* < 0.05). Additionally, significant increases in white blood cells, C-reactive protein, procalcitonin, lactate dehydrogenase (LDH), alanine transaminase, aspartate transaminase, ferritin, erythrocyte sedimentation rate, and D-dimer levels were observed in the high-dose group (*P* < 0.05). Specifically, LDH and ferritin were markedly higher in the high-dose group, with levels at 660.5 U/L and 475.05 ng/mL, respectively, compared to 450 U/L and 151.4 ng/mL in the medium-dose group, and 316.5 U/L and 120.5 ng/mL in the low-dose group. Correlation analysis indicated that LDH and ferritin levels were significantly and positively correlated with glucocorticoid dose (Spearman ρ = 0.672 and ρ = 0.654, respectively; *P* < 0.001).

**Conclusions:**

Serum LDH and ferritin levels may be useful biomarkers for determining the appropriate corticosteroid dosage in treating children with RMPP.

## Introduction

*Mycoplasma pneumoniae* (MP) is a common pathogen causing community-acquired pneumonia (CAP) in children. Typically, these infections are self-limiting and effectively treated with macrolide antibiotics. However, increasing cases of comorbidities and drug resistance have led to a higher likelihood of some infections progressing to refractory or severe pneumonia, which can be life-threatening [1, 2]. The severity of MP infection may correlate with the intensity of the host immune response. Consequently, immunosuppressive therapy, alongside appropriate antibiotic treatment, is beneficial for children with refractory *Mycoplasma pneumoniae* pneumonia (RMPP) [3]. Prior research has demonstrated that corticosteroids effectively manage MP infections by moderating aberrant immune responses, offering significant benefits to patients with RMPP. Nevertheless, the most effective treatment regimen has yet to be determined [4, 5].

Numerous studies have identified biomarkers such as D-dimer, lactate dehydrogenase (LDH), and ferritin (FER) to correlate with the severity of RMPP in children. Specifically, D-dimer levels have been positively associated with the severity of MP pneumonia (MPP) [6–8]. Additionally, serum LDH and FER levels are effective predictors of the condition’s severity and the necessary corticosteroid therapy for children with RMPP [9]. Despite these findings, few studies have focused on the correlation between these biomarkers and corticosteroid dosages in children with RMPP. Therefore, this study aims to explore the clinical significance of various biomarkers at different glucocorticoid therapy doses for RMPP in children and to provide guidance for clinical corticosteroid management.

## Methods

### Patients

We retrospectively collected clinical data from patients with MPP admitted to a children’s hospital between November 2017 and January 2022. This study adhered to the ethical standards of the Declaration of Helsinki (as revised in 2013).

The diagnosis of MPP was based on the following criteria: (1) clinical symptoms, signs, or radiographic findings indicative of pneumonia upon admission; (2) confirmation of MP infection by at least one of the following methods: a single serum anti-MP IgM ≥ 1:160, a four-fold increase or decrease in anti-MP IgM titer between the acute and recovery stages, or positive MP polymerase chain reaction results [10]. RMPP was diagnosed when patients, despite receiving appropriate antibiotic therapy for 7 days, continued to show exacerbated clinical signs, persistent fever, and progressive pulmonary imaging [2].

The inclusion criteria for the study were: (1) patients who met the diagnostic criteria for RMPP; (2) those who received corticosteroid treatment; and (3) those with complete hospitalization records [1, 11].

The exclusion criteria were: (1) co-infection with other pathogenic organisms; (2) incomplete hospitalization records; (3) prior corticosteroid treatment before admission; (4) corticosteroid therapy for other conditions such as asthma, congenital bronchopulmonary dysplasia, non-infectious interstitial pulmonary disease, and skin rash; and (5) underlying conditions such as asthma, chronic cardiac or pulmonary diseases, rheumatic diseases, and immunodeficiency.

### Study design

Based on prior studies and clinical observations, we categorized the 206 subjects into three groups: low-dose (*n* = 78), medium-dose (*n* = 84), and high-dose (*n* = 42). The low-dose group received intravenous methylprednisolone at ≤ 2 mg/kg/day (including 2 mg/kg/day), the medium-dose group received 2–10 mg/kg/day (with methylprednisolone dosage strictly between 2 mg/kg/day and 10 mg/kg/day), and the high-dose group received ≥ 10 mg/kg/day (including 10 mg/kg/day) [12–16].

After being diagnosed with MPP, all patients were initially treated with azithromycin at 10 mg/kg/day, administered orally once daily. If there was no improvement or if symptoms of pneumonia worsened after 3 days, we switched to doxycycline (2 mg/kg/day, administered twice daily) for those with macrolide-resistant MPP [2]. Additionally, all patients diagnosed with RMPP initially received low-dose methylprednisolone intravenously. If patients continued to show persistent fever, no improvement, or deterioration of clinical symptoms after 48 h of initial steroid therapy, we incrementally increased the dose of methylprednisolone, up to a maximum of 30 mg/kg/day. The patients were then divided into three groups based on the peak methylprednisolone dose administered [12, 13]. Patients were considered responsive to corticosteroid therapy if they experienced defervescence and an improvement in clinical symptoms or pulmonary lesions within 48 h after each treatment escalation. All patients ultimately recovered and were discharged from the hospital without any fatalities.

### Ethical statement

The study was approved by the Ethics Committee of Tianjin Children’s Hospital (No. 2021-KY-06) and was conducted by the Declaration of Helsinki guidelines. Because the study was retrospective and the data were anonymized, the Ethics Committee of Tianjin Children’s Hospital waived the requirement for informed consent.

### Data collection

The collection of clinical data from subjects was completed within 24 h of their diagnosis with RMPP and included the following components: (1) clinical characteristics such as age, sex, and BMI; (2) hypoxia, defined as an oxygen saturation level in room air < 92% [8]; (3) laboratory tests, which included white blood cells (WBC), C-reactive protein (CRP), procalcitonin (PCT), serum lactate dehydrogenase (LDH), alanine transaminase (ALT), aspartate transaminase (AST), FER, erythrocyte sedimentation rate (ESR), and D-dimer; (4) imaging examination findings, specifically pulmonary consolidation and pleural effusion; and (5) details of corticosteroid therapy.

### Statistical analysis

For data analysis, we used SPSS version 26.0. Continuous data were expressed as the median and interquartile range (IQR) and analyzed using the Mann-Whitney U test, the Kruskal-Wallis H test (K-W-H), and analysis of variance (ANOVA). Categorical data were presented as numbers (%) and analyzed using the chi-square test. Ordered multi-categorical data were analyzed using the K-W-H test. Correlations between variables were assessed using Spearman’s rank correlation coefficient. A *P*-value < 0.05 was considered statistically significant. Graphs were generated using GraphPad Prism version 9.0.

## Results

### Patient characteristics

During the study period, approximately 7600 children were hospitalized with MPP, and the incidence of RMPP was about 10.1%. A total of 206 patients were finally enrolled in this study. The distribution among the dosage groups was as follows: 78 patients in the low-dose group, 84 in the medium-dose group, and 42 in the high-dose group. The patient characteristics for each group are detailed in Table 1. In the low-dose group, the median age was 7.0 years (range 6.0–9.0), and the median BMI was 16.12 (range 14.50–18.96). The medium-dose group had a median age of 5.5 years (range 4.0–7.0) and a median BMI of 14.86 (range 13.83–16.51). The high-dose group’s median age was 6.0 years (range 4.0–7.0), and the median BMI was 14.58 (range 13.72–15.45) (*P* < 0.001). Significant differences were observed in both age and BMI across the three groups (*P* < 0.001), whereas there was no significant difference in sex distribution (*P* = 0.748). Additionally, the length of hospital stay was longest in the high-dose group at 13 days (range 10–15.25) (*P* < 0.001), and the incidence of hypoxemia also significantly varied among the groups (*P* < 0.001).

**Table 1:** Demographic and laboratory parameters of all study subjects

	Low-dose group (*n* = 78)	Medium-dose group (*n* = 84)	High-dose group (*n* = 42)	*P*-value
Age, years	7.0 (6.0 ~ 9.0)	5.5 (4.0 ~ 7.0) ^a*^	6.0 (4.0 ~ 7.0) ^b*^	< 0.001
BMI	16.12 (14.50 ~ 18.96)	14.86 (13.83 ~ 16.51) ^a*^	14.58 (13.72 ~ 15.45) ^b*^	< 0.001
Male, n (%)	44.9%	42.9%	50%	0.748
Length of stay, days	7(6 ~ 8)	8(7 ~ 9.75) ^a*^	13 (10 ~ 15.25) ^b*c*^	< 0.001
Hypoxemia, n (%)	32(41%)	64 (76.2%) ^a*^	41 (97.6%) ^b*c*^	< 0.001
Laboratory parameters				
WBC (×10^9^/L)	7.94 (6.74 ~ 10.98)	11.59 (8.4 ~ 14) ^a*^	13.64 (11.15 ~ 18.36) ^b*c*^	< 0.001
CRP (mg/L)	16.12 (14.50 ~ 18.96)	31.85 (18 ~ 59.8) ^a*^	59 (38.1 ~ 97) ^b*c*^	< 0.001
PCT (ng/mL)	0.09 (0.06 ~ 0.14)	0.19(0.1 ~ 0.47) ^a*^	0.48(0.26 ~ 0.88) ^b*c*^	< 0.001
LDH (U/L)	316.5 (272.75 ~ 381.5)	450 (355 ~ 589.5) ^a*^	660.5 (532 ~ 806.5) ^b*c*^	< 0.001
ALT (U/L)	14.00 (10.00 ~ 19.00)	14.5 (12 ~ 21)	20 (11.75 ~ 31.5) ^b*c*^	0.005
AST (U/L)	26(23 ~ 31)	34.5 (27.25 ~ 41.5) ^a*^	41 (30.75 ~ 58) ^b*c*^	< 0.001
FER (ng/mL)	120.5 (81 ~ 142.7)	151.4 (118.18 ~ 275.9)^a*^	475.05 (313.98 ~ 694.23)^b*c*^	< 0.001
ESR (mm/h)	27 (22.75 ~ 36)	29.5(20 ~ 41)	36(25.5 ~ 51.25) ^b*c*^	0.007
D-dimer (µg/L)	0.1 (0.1 ~ 0.3)	0.25(0.1 ~ 0.5) ^a*^	1.1(0.2 ~ 2.9) ^b*c*^	< 0.001

Continuous data are expressed as median (IQR), and categorical data are expressed as the number of patients (%). *P*-value < 0.05 was considered statistically significant. *P < 0.001. ^a^compared between the low-dose group and the medium-dose group. ^b^compared between the low-dose group and the high-dose group. ^c^compared between the medium-dose group and the high-dose group. BMI: body mass index. WBC: white blood cell; CRP: C-reactive protein; procalcitonin: PCT; lactate dehydrogenase: LDH; ALT: alanine aminotransferase; AST: aspartate aminotransferase; FER: ferritin; ESR: erythrocyte sedimentation rate

### Laboratory findings

**Fig. 1:**
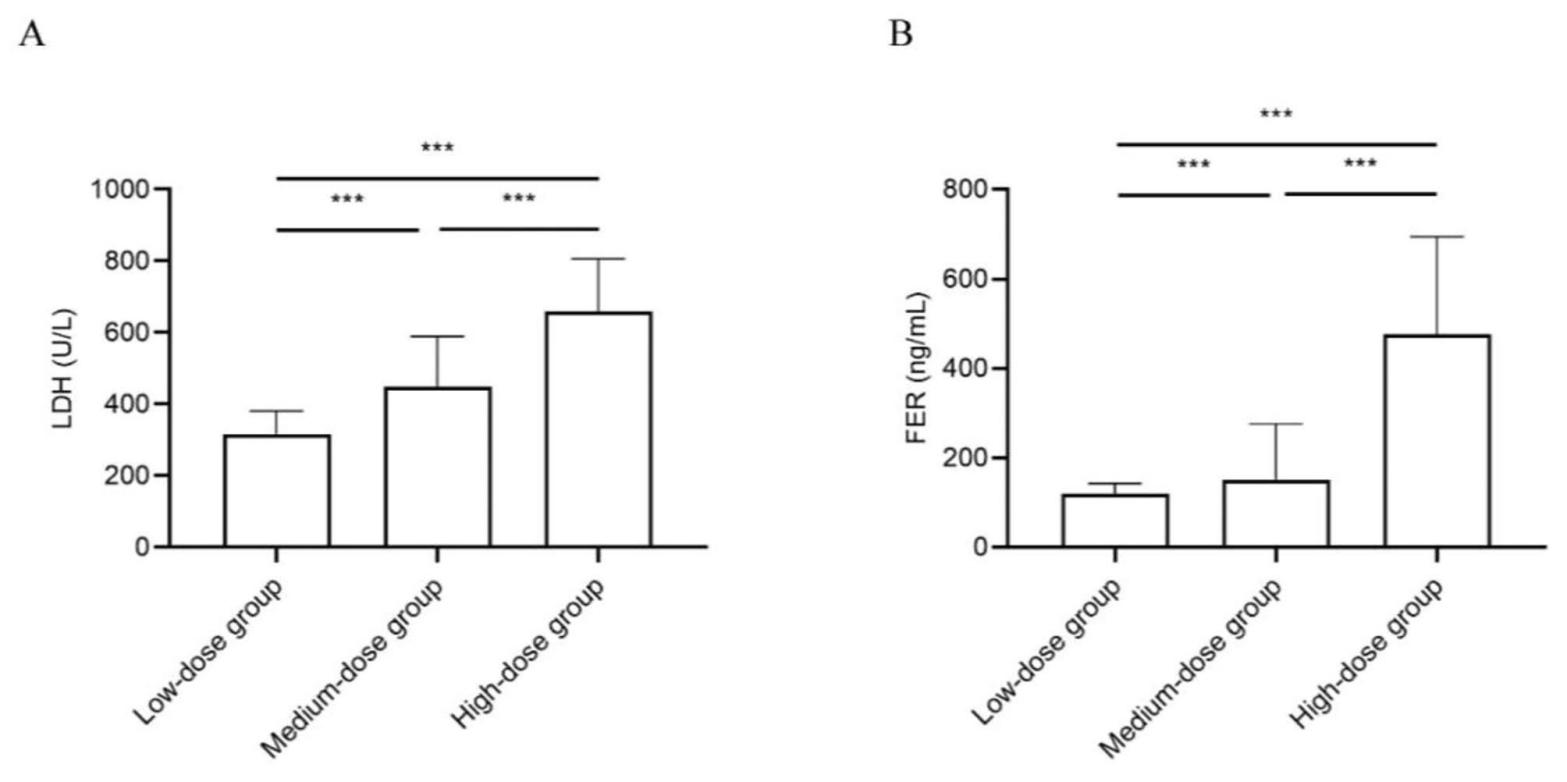
Comparative analysis of ferritin levels among low-dose group, medium-dose group, and high-dose group (*denotes statistical significance, and whiskers indicate median with interquartile range). (**A**) shows that LDH level (U/L) was significantly higher in the high-dose group compared to the medium-dose group (median 660.5 vs. 450 ng/mL, *P* < 0.001***) and the low-dose group (median 450 vs. 316.5 ng/mL, *P* < 0.001***). (**B**) shows that ferritin level (ng/mL) was significantly higher in the high-dose group compared to the medium-dose group (median 475.05 vs. 151.4 ng/mL, *P* < 0.001***) and the low-dose group (median 475.05 vs. 120.5 ng/mL, *P* < 0.001***). The Mann-Whitney U-test was used to calculate the *P*-value. LDH, lactate dehydrogenase; FER, ferritin

The laboratory findings for the patients are detailed in Table 1. Patients with RMPP in the high-dose group exhibited higher levels of WBC, CRP, PCT, LDH, ALT, AST, FER, ESR, and D-dimer compared to the other groups (*P* < 0.01). Notably, LDH and FER levels were dramatically higher in the high-dose group than in the low-dose and medium-dose groups (Fig. 1).

### Radiographic examinations

All subjects underwent chest X-rays or CT scans following admission. The radiographic features for the three groups are shown in Table 2. There were no significant differences in the incidence of pleural thickening among the groups. However, the incidence of pleural effusion (7.7% vs. 33.3% vs. 54.8%, *P* < 0.001) and atelectasis (19.6% vs. 34.5% vs. 47.6%, *P* = 0.004) was significantly higher in the high-dose group. Moreover, statistically significant differences were observed in the incidence of pulmonary consolidation among the groups (*P* = 0.013). Specifically, 28 patients (35.9%) in the low-dose group, 36 patients (42.9%) in the medium-dose group, and 28 patients (66.7%) in the high-dose group showed areas of pulmonary consolidation covering ≥ ½ of the lung.

**Table 2:** Radiographic imaging results of all study subjects

Parameter	Low-dose group (*n* = 78)	Medium-dose group (*n* = 84)	High-dose group (*n* = 42)	*P*-value
Area of pulmonary consolidation, n (%)				
0	1(1.3)	6 (7.1)	2 (4.8)	0.013
<1/2	49 (62.8)	42 (50.0)	12 (28.6)	
≥ 1/2	28 (35.9)	36 (42.9)	28 (66.7)	
Pleural effusion, n (%)	6 (7.7)	28 (33.3) ^a*^	23 (54.8) ^b*c*^	< 0.001
Lobar atelectasis, n (%)	15 (19.6)	29 (34.5) ^a*^	20 (47.6) ^b*^	0.004
Pleural thickening, n (%)	56 (71.8)	53 (63.1)	28 (66.7)	0.498

Categorical data are expressed as the number of patients (%). *P*-value < 0.05 was considered statistically significant. *P < 0.001. ^a^compared between the low-dose group and the medium-dose group. ^b^compared between the low-dose group and the high-dose group. ^c^compared between the medium-dose group and the high-dose group

### Correlation analysis of different variables

**Fig. 2:**
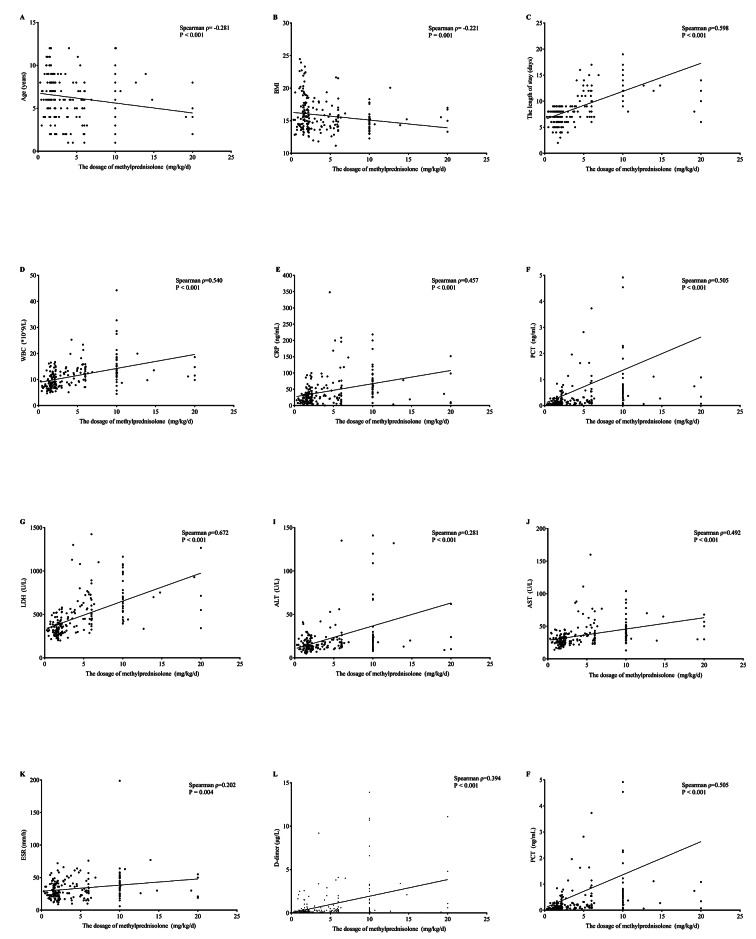
Correlation analysis between the dosage of methylprednisolone and other variables. LDH (Spearman ρ = 0.672) and FER (Spearman ρ = 0.654) had highest correlation coefficient with the dosage of methylprednisolone. WBC: white blood cell; CRP: C-reactive protein; PCT: procalcitonin; LDH: lactate dehydrogenase; ALT: alanine aminotransferase; AST: aspartate aminotransferase; FER: ferritin; ESR: erythrocyte sedimentation rate

We employed the Spearman correlation test to analyze the relationship between the methylprednisolone dosage and various clinical and laboratory variables. As shown in Fig. 2, the dosage of methylprednisolone demonstrated positive correlations with the length of hospital stay and the levels of WBC, CRP, PCT, LDH, ALT, AST, ESR, FER, and D-dimer (all *P* < 0.05). Interestingly, both age and BMI were negatively correlated with the dosage of methylprednisolone (Spearman ρ = -0.281 and ρ = -0.221, respectively). Among these variables, LDH and FER exhibited the highest correlation coefficients with methylprednisolone dosage (Spearman ρ = 0.672 and ρ = 0.654, respectively).

## Discussion

In this study, we found that glucocorticoid dosage was positively correlated with the levels of WBC, CRP, PCT, ALT, AST, ESR, and D-dimer and negatively correlated with age and BMI. Notably, LDH and ferritin FER exhibited the highest correlation coefficients among all biomarkers, at 0.674 and 0.657, respectively, suggesting a strong positive association with glucocorticoid dose in children with RMPP. These findings will assist pediatricians in assessing the severity of RMPP and selecting the appropriate glucocorticoid therapy dosage for affected children.

In recent years, there has been an increase in the recognition of RMPP. Researchers have emphasized the role of biomarkers in assessing the severity of RMPP [4, 17]. A previous study indicated that D-dimer levels could evaluate RMPP severity, with D-dimer levels ≤ 280 ng/ml potentially indicating mild pneumonia and a low incidence of pleural effusion [18]. Similarly, our findings show that the median D-dimer level in the low-dose group was 0.1 µg/L with a 7.7% incidence of pleural effusion. Furthermore, the medium-dose group had serum FER levels of 151.4 ng/mL, nearing levels that predict severe forms of MPP, suggesting that children with elevated FER levels may progress to more severe RMPP [19]. Additionally, prior research suggested that CRP levels ≥ 50 mg/L and LDH levels ≥ 480 U/L are predictors of delayed radiographic clearance [20]. Consistent with this, our high-dose group exhibited a CRP level of 59 mg/L, an LDH level of 660.5 U/L, and more severe radiographic manifestations. Together with previous reports, our results indicate that the severity of pneumonia in children with RMPP may be linked to levels of inflammatory biomarkers.

Although macrolides are the current first-line antibiotics, patients with RMPP may benefit from immunosuppressive therapy due to strong immunological reactions associated with their condition. Numerous researchers regard RMPP as an indication for corticosteroid use [4, 21]. Steroids help downregulate overactive immune responses, alleviate clinical symptoms and pulmonary injuries, and reduce complications in children and adults with MP infections [7, 21, 22]. There have been extensive reports on steroid regimens. This study indicated that the low-dose group exhibited relatively lower laboratory indices, such as CRP levels at 16.12 mg/L, and presented milder clinical signs, akin to findings in a study on RMPP children treated with methylprednisolone at 2 mg/kg/day [23]. However, Chen et al. have indicated that low-dose methylprednisolone may be ineffective for RMPP with extensive pulmonary consolidation [24]. Yang et al. suggested that intravenous methylprednisolone at doses of 5–10 mg/kg/day is more effective for severe cases [25]. Our findings also revealed that incidences of pulmonary consolidation covering more than half the lung and pleural effusion were higher in the medium-dose group, suggesting more severe pneumonia compared to the low-dose group. The high-dose group had the highest incidence of hypoxemia, lobar atelectasis, and the longest hospital stays [12, 14], supporting the notion that the dosage of corticosteroid therapy correlates with the severity of RMPP in children.

LDH is a cytoplasmic enzyme widely distributed across major organ systems. When cell lysis occurs or cell membranes are damaged, LDH is released into the extracellular space [6, 26]. Previous studies have suggested that LDH is an early indicator for the initiation of glucocorticoid therapy, with effective ranges from 302 to 480 U/L [2, 27, 28]. This aligns with our results, as the LDH level in the low-dose group was 316.5 U/L. Furthermore, the LDH level in the medium-dose group was 450 U/L, consistent with the findings by Chen et al., who demonstrated that when serum LDH levels reach ≥ 478 IU/L, low-dose methylprednisolone may be ineffective for RMPP [24].

FER is primarily synthesized by macrophages and often increases in response to tissue injury, pathogenic infections, and inflammation [29, 30]. Wen et al. showed that children with serum FER levels ≥ 329 ng/mL were more likely to have RMPP [31]. Kawamata et al. reported that FER levels of 291.5 ng/mL suggest that glucocorticoid therapy can be initiated in patients with MPP [9]; however, the FER levels in our study were higher. In our study, the median FER level in the low-dose group was 120.5 ng/mL. The reasons for this difference might be as follows: (1) it suggests that early and timely initiation of glucocorticoid therapy is necessary for patients with RMPP; (2) there may be regional and seasonal variations in the epidemiology of MP.

Our study’s correlation analysis revealed that the correlation coefficients for LDH and FER were 0.672 and 0.657, respectively, indicating a positive correlation with the doses of methylprednisolone in children with RMPP. Zheng et al. revealed that the association between LDH levels and the development of RMPP exhibits a non-linear dose-response relationship [32], consistent with our findings that LDH levels positively correlate with glucocorticoid doses. Zhu et al. found that LDH levels ≥ 590 IU/L and FER levels ≥ 411 ng/mL might be significant clinical markers in RMPP patients treated with pulse-dose glucocorticoids, which were relatively lower than our results [11]. In our study, the LDH and FER levels in the high-dose group were 660.5 U/L and 475.05 ng/mL, respectively. This discrepancy could be explained by the fact that Zhu et al. defined pulse-dose methylprednisolone as 200 mg/day, whereas our study defined high-dose methylprednisolone as 10 mg/kg/day. We concluded that LDH and FER are useful biomarkers for determining the appropriate dosage of glucocorticoids in treating children with RMPP.

This study has several limitations. First, it was not a randomized controlled trial, making it susceptible to bias from potential confounding variables. Second, selection bias may have initially resulted from differences in the timing of corticosteroid administration. Finally, this was a small-scale, single-center, retrospective study, and larger, multi-center, prospective studies are needed to further validate these results.

## Conclusions

In summary, LDH and ferritin levels are associated with the severity of the inflammatory response in Mycoplasma pneumonia and may serve as biomarkers to determine the appropriate dosage of glucocorticoids. These findings could provide a theoretical basis for physicians to guide glucocorticoid administration in children with RMPP.

## Data Availability

The datasets generated and/or analysed during the current study are not publicly available due [REASON WHY DATA ARE NOT PUBLIC] but are available from the corresponding author on reasonable request.

## Abbreviations

ALT: Alanine aminotransferase
AST: Aspartate aminotransferase
BMI: Body mass index
CAP: Community-acquired pneumonia
CRP: C-reactive protein
ESR: Erythrocyte sedimentation rate
FER: Ferritin
LDH: Lactate dehydrogenase
MP: Mycoplasma pneumoniae
MPP: Mycoplasma pneumoniae pneumonia
PCT: Procalcitonin
RMPP: Refractory Mycoplasma pneumoniae pneumonia
WBC: White blood cell
